# Association between circulating selenium levels and arterial stiffness: a nationwide cross-sectional study

**DOI:** 10.1097/JS9.0000000000003400

**Published:** 2025-09-23

**Authors:** Kunsheng Zhao, Lei Qiao, Wenqi Hu, Wenhai Sui, Xiuchang Li, Guang Zhang

**Affiliations:** a Department of Health Management, Shandong Engineering Research Center of Health Management, The First Affiliated Hospital of Shandong First Medical University & Shandong Provincial Qianfoshan Hospital, Shandong Institute of Health Management, Jinan, People’s Republic of China; b National Key Laboratory for Innovation and Transformation of Luobing Theory; The Key Laboratory of Cardiovascular Remodeling and Function Research, Chinese Ministry of Education, Chinese National Health Commission and Chinese Academy of Medical Sciences; Department of Cardiology, Qilu Hospital of Shandong University, Jinan, People’s Republic of China; c Department of Cardiology, The Second Affiliated Hospital of Shandong First Medical University, Taian, People’s Republic of China

**Keywords:** arterial stiffness, AIP, ePWV, selenium

## Abstract

**Background:**

Atherosclerotic cardiovascular disease (ASCVD) is the leading cause of death worldwide. Arterial stiffness is an important pathological characteristic of ASCVD. Selenium (Se) may have a notable effect of arterial stiffness. However, the relationship between circulating Se (CSe) levels and arterial stiffness remains unclear.

**Methods:**

A total of 11 937 participants from the National Health and Nutrition Examination Survey (NHANES) from 2011 to 2018 were enrolled. The relationship between CSe levels, estimated pulse wave velocity (ePWV), and the atherogenic index of plasma (AIP) was evaluated using univariate and multivariate linear regression, dose-response, and mediation analyses.

**Results:**

A linear relationship between the CSe index and high AIP (*P* for nonlinearity = 0.1) and a U-shaped relationship between the CSe index and high ePWV were found (*P* for nonlinearity < 0.001). In fully adjusted models, the relationship between CSe and high AIP was positive (OR = 1.48, 95% CI: 1.20, 1.82; *P* = 0.001). An inflection point was found in the relationship between the CSe index and high ePWV. CSe levels of < 2.99 μmol/L were negatively related to high ePWV (OR = 0.71; 95% CI: 0.59, 0.86; *P* < 0.001). In contrast, CSe levels of ≥ 2.99 μmol/L were positively related to high ePWV (OR = 2.66; 95% CI: 1.66, 4.25; *P* < 0.001). Furthermore, the indirect effect mediated by the sex of CSe on high AIP was prominent (indirect effect = 0.006; 95% CI: 0.003, 0.008; percent mediation = 26.49%) in the fully adjusted models.

**Conclusions:**

Our findings suggests a significant relationship between CSe levels and arterial stiffness, the mechanism behind which should be further explored.

**HIGHLIGHTS**

- This is the first study to comprehensively demonstrate the relationship between CSe levels and arterial stiffness in the population representing the whole country.
- CSe levels and AIP were positively correlated with a linear trend, while CSe levels and ePWV were correlated with a U-shaped trend.
- Sex plays a certain mediating role in the relationship between CSe levels and arterial stiffness.

## Introduction

Atherosclerotic cardiovascular disease (ASCVD) is a leading cause of global death^[1]^. In recent years, the incidence of ASCVD in youth has increased^[2–4]^. Arterial stiffness is an important pathological characteristic in ASCVD. The standard method for measuring arterial stiffness is the carotid-femoral pulse wave velocity (cfPWV); the greater the cfPWV, the worse the vascular elasticity^[5]^. However, owing to the complexity of this procedure, the cfPWV has not been widely promoted in clinical practice. To solve this problem, estimated pulse wave velocity (ePWV) is used clinically as an alternative to cfPWV. ePWV, calculated based on age and mean blood pressure (MBP), is an effective indicator of cfPWV and has good consistency with in vivo evaluation^[6]^.

The atherogenic index of plasma (AIP), a predictor of lipid metabolism disorders, is regarded as a more superior predictor of cardiovascular disease (CVD) than the ordinary predictors^[7,8]^. AIP is related to various diseases such as hypertension, sleep quality, degeneration of bone microstructure, and severity of viral infections^[9–11]^. Several studies have shown that the AIP is a powerful marker of CVD risks^[8,12,13]^. A population-based national cohort study revealed that AIP is still observably related to cardiovascular risk after adjusting for numerous traditional risk factors^[14]^, and is an independent predictor of the development of cardiovascular events and their associated mortality^[15]^. In addition, compared to traditional risk factors, AIP showed a superior advantage in predicting rapid plaque progression^[16]^.

Selenium (Se), an important essential heavy metal microelement, is involved in many important physiological activities including glucolipid consumption, immunoreaction, antiinflammation, and antioxidation^[17–19]^. As is well-known, abnormal lipid metabolism, oxidative stress (OS), immune responses, and inflammation are key factors in the pathogenesis of arterial stiffness^[20,21]^. Thus, Se may have a significant effect on arterial stiffness. Nevertheless, the correlation between circulating Se (CSe) levels and arterial stiffness remains unclear. Our study aimed to clarify this relationship through a population-based cross-sectional study

## Materials and methods

### Study population

Participants were recruited from the National Health and Nutrition Examination Survey (NHANES) project that was executed among the general population by the Centers for Disease Control and Prevention (CDC) to evaluate the health status of US population. Written informed consent was obtained from all the participants before the investigation began. The survey was approved by the Institutional Review Board of the CDC (protocol code: 2011-17; 2018-01). Therefore, an additional ethical review of our study was exempted. This cross-sectional study has been reported in line with the strengthening of the reporting of cohort, cross-sectional, and case-control studies (STROCSS) guidelines^[22]^. In this cross-sectional study, we included participants (*n* = 39 156) from surveys conducted over four cycles of the NHANES from 2011 to 2018. After application of inclusion criteria, 27 219 individuals were unqualified: age < 20 years (*n* = 16 539), cancer (*n* = 2184), pregnancy (*n* = 245), missing values for CSe, systolic blood pressure (SBP), triglyceride (TG), and high-density lipoprotein (HDL) (*n* = 8249), and abnormal values (*n* = 2). Ultimately 11 937 individuals were analyzed in this study. Details of the screening flowchart are presented in Supplementary Digital Content, Fig. S1, available at: http://links.lww.com/JS9/F16

### AIP and ePWV calculation

The AIP was calculated from TG and HDL levels based on the equation^[23]^: AIP = Log_10_ (TG/HDL). The ePWV index was calculated from age and blood pressure based on the equation^[24]^: ePWV = 9.587 − 0.402 × age + 4.560 × 10^−3^ × age^2^ − 2.621 × 10^−5^ × age^2^ × MBP + 3.176 × 10^−3^ × age × MBP − 1.832 × 10^−2^ × [diastolic blood pressure (DBP) + 0.4 × (SBP − DBP)].

### Case definition

The diagnosis of diseases is initially determined based on the disease diagnosis history or medication use history in the questionnaire. If the respondent answers “Yes,” the participant is identified as having the disease. In addition, it is confirmed based on the corresponding examination indicators. To reduce errors, the final blood pressure value is based on the average of three consecutive measurements. If the respondent’s SBP is higher than 130 mmHg and/or the DBP is higher than 80 mmHg, the participant is identified as a hypertensive patient^[25]^. If the respondent’s fasting blood glucose (FBG) is higher than 7.0 mmol/L and/or HbA1c is higher than 6.5% and/or the blood glucose 2 hours after the glucose tolerance test is higher than 11.1 mmol/L, the participant is identified as a diabetic patient^[26]^.

### Variates assessment

Age, sex, and race were obtained from the demographic module. Family poverty ratio of income (FPRI), education levels, status of marriage, smoking, and drinking were obtained from the demographic module. Total cholesterol (TC), TG, HDL, low-density lipoprotein (LDL), FBG, and CSe were obtained from the laboratory module. SBP, DBP, and body mass index (BMI) were obtained from the examination module. Se and energy intake were obtained from the dietary module.

### Statistical analysis

According to the NHANES guidelines, appropriate sample weights were adopted in the analysis to represent the national population in the United States. Continuous variables with normal distributions were expressed as the mean ± standard error (SE). Categorical variables were presented as numbers (%). Differences in continuous variables were analyzed using t tests or analysis of variance (ANOVA), and chi-squared tests were applied to evaluate differences in categorical variables. Univariate logistic regression analysis was used to reveal the correlation between various variables and the arterial stiffness index. The selection of covariates was determined based on the results of univariate regression analysis, with a reference *P* value < 0.05. Multivariate logistic analysis was utilized in three adjusted models to explore the independent correlation between CSe levels and arterial stiffness index. Model 1 represents a rough analysis without any variable adjustment. Model 2 is a slightly corrected analysis, with adjustments made for three variables: age, sex, and race. Model 3 was further analyzed through correction, adjusting for age, sex, race, education, marital status, smoking, drinking, hypertension, diabetes, CHD, stroke, antihypertensive drugs, antidiabetic drugs, TC, LDL, SBP, DBP, BMI, FBG, Se intake, and energy intake. AIP was analyzed not only as a categorical variable according to the third quartile, but also as a continuous variable. Univariate and multivariate linear regressions were also applied when the AIP was treated as a continuous variable. The dose-effect correlation was analyzed in the fully adjusted models using a restricted cubic spline. If the relationship was nonlinear, the inflection point would be calculated using a segmented regression model, and then constructed a two-piecewise logistic regression analysis based on the inflection point. Subgroup analyses were performed to detect the interaction variables. The impact of intermediary variables on the relationships was calculated by mediation analysis using the product of coefficients method. Sensitivity analysis was used to test the stability of the results when AIP was regarded as continuous values. Furthermore, we also compared the results of the data before and after imputation to observe the influence of missing values and imputation on the results. High AIP or ePWV was defined as the third digit in the AIP or ePWV quartiles. Multicollinearity was examined using variance inflation factors (VIF). There were multicollinearity problems in our analysis, with all VIF values of the variables less than 5^[27]^. Missing covariate values were filled using multiple imputation based on chained equations using a “mice” package. All statistical analyses were completed with R software (version 4.4.2; The R Foundation for Statistical Computing), and *P* ≤ 0.05 for a two-tailed test was considered statistically significant.

## Results

### Characteristics of the study participants

In this study, 11 937 participants (5988 males and 5949 females) were included in the analysis. The mean age was 46.19 ± 0.39 years. The baseline demographic characteristics of the four quartiles as defined by AIP are presented in Table 1. Compared with the lowest quartile of the AIP index, participants in the higher quartiles of the AIP index were more likely to be older, male, non-Hispanic white, with lower FPRI, less educated, married, smoker, less drinking, hypertension, diabetes, CHD, stroke, taking antihypertensive drugs, taking antidiabetic drugs, hyperlipidemia, lower HDL, and higher levels of BP, BMI, FBG, CSe, Se intake, and energy intake. Similar results are presented for the participants according to ePWV index quartiles in Supplementary Digital Content, Table S1, available at: http://links.lww.com/JS9/F17. Compared with the lowest quartile of ePWV index, individuals with a higher quartile of the ePWV index tended to be older, male, non-Hispanic white, poor, less educated, married, smoker, less drinking, hypertension, diabetes, CHD, stroke, taking antihypertensive drugs, taking antidiabetic drugs, hyperlipidemia, less energy intake, and higher levels of BP, BMI, FBG, CSe, and Se intake.

**Table 1:** Baseline characteristics of the participants by quartiles of AIP index

		AIP				
Variable	Overall	Q1	Q2	Q3	Q4	*P* value
		(<—0.228)	(–0.228–0.002)	(0.002–0.242)	(≥ 0.242)	
No. of participants	11 937	2984	2983	2984	2986	
Age (years)	46.19 ± 0.39	44.11 ± 0.49	45.81 ± 0.55	47.42 ± 0.51	47.48 ± 0.45	< 0.001
						< 0.001
Young	5309 (47.90)	1567 (53.26)	1337 (49.41)	1195 (44.93)	1210 (43.84)	
Middle	3028 (28.84)	643 (25.85)	717 (26.11)	775 (29.58)	893 (33.93)	
Older	3600 (23.26)	774 (20.89)	929 (24.49)	1014 (25.49)	883 (22.23)	
Sex (*n*, %)						< 0.001
Male	5988 (50.05)	1096 (34.76)	1356 (44.98)	1553 (52.10)	1983 (68.81)	
Female	5949 (49.95)	1888 (65.24)	1627 (55.02)	1431 (47.90)	1003 (31.19)	
Race (*n*, %)						< 0.001
Mexican American	1637 (9.23)	250 (6.19)	382 (8.62)	460 (10.38)	545 (11.81)	
Other Hispanic	1285 (6.79)	258 (6.24)	282 (6.04)	354 (6.78)	391 (8.14)	
Non-Hispanic White	4148 (63.37)	965 (62.41)	1011 (63.58)	1048 (63.15)	1124 (64.37)	
Non-Hispanic Black	2765 (11.32)	1014 (16.92)	803 (12.88)	580 (9.31)	368 (5.98)	
Other	2102 (9.30)	497 (8.24)	505 (8.88)	542 (10.39)	558 (9.71)	
FPRI (n, %)						0.004
< 1	2489 (14.43)	583 (13.62)	627 (14.25)	604 (13.98)	675 (15.86)	
1–3	5272 (38.32)	1248 (34.98)	1335 (39.42)	1345 (40.45)	1344 (38.51)	
> 3	4176 (47.26)	1153 (51.40)	1021 (46.33)	1035 (45.57)	967 (45.63)	
Education (*n*, %)						< 0.001
<High school	2606 (14.05)	489 (10.23)	623 (12.71)	706 (16.29)	788 (17.08)	
High school	2697 (23.06)	628 (19.58)	697 (23.77)	675 (24.23)	697 (24.73)	
>High school	6634 (62.90)	1867 (70.20)	1663 (63.53)	1603 (59.48)	1501 (58.19)	
Marital status (*n*, %)						< 0.001
Married	5949 (53.37)	1284 (48.84)	1430 (50.86)	1560 (55.41)	1675 (58.54)	
Never	2466 (20.43)	848 (25.79)	632 (22.38)	550 (18.72)	436 (14.65)	
Other	3522 (26.20)	852 (25.38)	921 (26.76)	874 (25.87)	875 (26.81)	
Smoking (*n*, %)						< 0.001
Never	6929 (57.19)	1905 (63.01)	1808 (59.97)	1686 (55.32)	1530 (50.29)	
Ever	2662 (23.94)	561 (21.45)	614 (22.01)	705 (24.99)	782 (27.41)	
Current	2346 (18.86)	518 (15.54)	561 (18.02)	593 (19.69)	674 (22.30)	
Drinking (*n*, %)						< 0.001
Never	2298 (15.41)	411 (10.36)	561 (15.10)	644 (17.25)	682 (19.06)	
Low-middle	8675 (74.26)	2292 (77.23)	2171 (74.23)	2125 (73.73)	2087 (71.76)	
Heavy	964 (10.34)	281 (12.41)	251 (10.66)	215 (9.02)	217 (9.19)	
Hypertension (*n*, %)						< 0.001
No	5622 (52.09)	1720 (63.80)	1469 (55.38)	1307 (48.78)	1126 (40.05)	
Yes	6315 (47.91)	1264 (36.20)	1514 (44.62)	1677 (51.23)	1860 (59.95)	
Diabetes (*n*, %)						< 0.001
No	9708 (86.69)	2704 (94.23)	2536 (90.75)	2328 (84.48)	2140 (77.05)	
Yes	2229 (13.31)	280 (5.77)	447 (9.25)	656 (15.52)	846 (22.95)	
CHD (*n*, %)						< 0.001
No	11,528 (97.07)	2919 (98.15)	2910 (98.12)	2859 (96.57)	2840 (95.41)	
Yes	409 (2.93)	65 (1.85)	73 (1.88)	125 (3.43)	146 (4.59)	
Stroke (*n*, %)						0.012
No	11,526 (97.58)	2897 (97.87)	2890 (98.21)	2868 (97.42)	2871 (96.82)	
Yes	411 (2.42)	87 (2.13)	93 (1.79)	116 (2.58)	115 (3.18)	
Antihypertensive drugs (*n*, %)						< 0.001
No	7084 (64.16)	2024 (73.47)	1816 (66.72)	1677 (60.72)	1567 (55.43)	
Yes	4853 (35.84)	960 (26.53)	1167 (33.28)	1307 (39.29)	1419 (44.57)	
Antidiabetic drugs (*n*, %)						< 0.001
No	11,482 (97.45)	2927 (98.63)	2894 (98.10)	2866 (97.54)	2795 (95.51)	
Yes	455 (2.55)	57 (1.37)	89 (1.90)	118 (2.46)	191 (4.49)	
TC (mmol/L)	4.94 ± 0.02	4.73 ± 0.026	4.84 ± 0.024	4.95 ± 0.028	5.27 ± 0.031	< 0.001
TG (mmol/L)	1.67 ± 0.03	0.72 ± 0.01	1.12 ± 0.01	1.63 ± 0.01	3.23 ± 0.04	< 0.001
HDL (mmol/L)	1.38 ± 0.01	1.79 ± 0.01	1.45 ± 0.01	1.25 ± 0.01	1.03 ± 0.01	< 0.001
LDL (mmol/L)	2.08 ± 0.03	2.25 ± 0.02	2.14 ± 0.03	2.05 ± 0.04	1.88 ± 0.05	0.001
SBP (mmHg)	121.71 ± 0.29	118.28 ± 0.41	121.24 ± 0.45	122.51 ± 0.47	124.92 ± 0.38	< 0.001
DBP (mmHg)	71.36 ± 0.29	69.23 ± 0.37	70.81 ± 0.40	71.70 ± 0.37	73.78 ± 0.36	< 0.001
BMI (kg/m^2^)	29.24 ± 0.14	26.13 ± 0.18	28.64 ± 0.18	30.60 ± 0.20	31.67 ± 0.20	< 0.001
FBG (mmol/L)	5.52 ± 0.02	5.08 ± 0.02	5.23 ± 0.03	5.58 ± 0.04	6.19 ± 0.08	< 0.001
CSe (μmol/L)	2.47 ± 0.01	2.43 ± 0.01	2.45 ± 0.01	2.48 ± 0.01	2.53 ± 0.01	< 0.001
Se intake (μg/d)	132.12 ± 3.51	124.22 ± 1.90	126.95 ± 2.51	142.15 ± 1.32	135.47 ± 2.43	< 0.001
Energy intake (kal/d)	1974.96 ± 11.73	1869.64 ± 19.81	1947.89 ± 24.47	1965.48 ± 20.66	2119.70 ± 22.58	< 0.001

AIP, atherogenic index of plasma; BMI, body mass index; CHD, coronary heart disease; CSe, circulating selenium; DBP, diastolic blood pressure; FBG, fasting blood glucose; FPRI, family poverty ratio of income; HDL, high-density lipoprotein; LDL, low-density lipoprotein; SBP, systolic blood pressure; TC, total cholesterol; TG, triglyceride.

### CSe and the risk of high AIP and high ePWV

The correlation test for each variable and high AIP is shown in Table 2. The age, smoking, hypertension, T2DM, CHD, stroke, FBG, use of antihypertensive drugs, use of antidiabetic drugs, TC, SBP, DBP, BMI, FBG, CSe, Se intake, and energy intake were positively associated with AIP. Nevertheless, sex, race, education, marital status, drinking, and LDL were negatively related to the AIP (all *P* < 0.05). The FPRI was not associated with AIP (*P* > 0.05). The correlation between the covariants and ePWV explored in the univariate logistic regression analysis is shown in Supplementary Digital Content, Table S2, available at: http://links.lww.com/JS9/F17. Similar results revealed that sex, race, FPRI, marital status (other), smoking status (ever), hypertension, T2DM, CHD, stroke, use of antihypertensive drugs, use of antidiabetic drugs, TC, TG, HDL, BMI, and FBG levels were positively related to ePWV. However, education, marital status (never), smoking status (current), drinking, CSe, and energy intake were negatively related to ePWV (all *P* < 0.05). LDL, BMI, and Se intakes were not related to ePWV (all *P* > 0.05).

**Table 2:** Univariate logistic regression analysis of high AIP

Variable	OR (95% CI)	*P* value
Age (years)	1.01 (1.00, 1.01)	< 0.001
Sex (*n*, %)		
Male	Ref	
Female	0.35 (0.32, 0.40)	< 0.001
Race (*n*, %)		
Mexican American	Ref	
Other Hispanic	0.91 (0.76, 1.08)	0.282
Non-Hispanic White	0.72 (0.62, 0.85)	< 0.001
Non-Hispanic Black	0.32 (0.27, 0.39)	< 0.001
Other	0.75 (0.63, 0.89)	0.002
FPRI (*n*, %)		
<1	Ref	
1–3	0.89 (0.76, 1.03)	0.122
>3	0.84 (0.69, 1.02)	0.080
Education (*n*, %)		
<High school	Ref	
High school	0.84 (0.67, 1.06)	0.141
>High school	0.69 (0.59, 0.81)	< 0.001
Marital status (*n*, %)		
Married	Ref	
Never	0.58 (0.49, 0.69)	< 0.001
Other	0.91 (0.80, 1.04)	0.160
Smoking (*n*, %)		
Never	Ref	
Ever	1.42 (1.18, 1.71)	< 0.001
Current	1.49 (1.30, 1.70)	< 0.001
Drinking (*n*, %)		
Never	Ref	
Low-middle	0.71 (0.63, 0.81)	< 0.001
Heavy	0.64 (0.49, 0.83)	0.002
Hypertension (*n*, %)		
No	Ref	
Yes	1.91 (1.70, 2.14)	< 0.001
Diabetes (*n*, %)		
No	Ref	
Yes	2.64 (2.18, 3.19)	< 0.001
CHD (*n*, %)		
No	Ref	
Yes	1.97 (1.40, 2.79)	< 0.001
Stroke (*n*, %)		
No	Ref	
Yes	1.49 (1.12, 1.97)	0.008
Antihypertensive drugs (*n*, %)		
No	Ref	
Yes	1.64 (1.43, 1.88)	< 0.001
Antidiabetic drugs (*n*, %)		
No	Ref	
Yes	2.42 (1.78, 3.30)	< 0.001
TC (mmol/L)	1.49 (1.40, 1.59)	< 0.001
LDL (mmol/L)	0.75 (0.68, 0.83)	< 0.001
SBP (mmHg)	1.02 (1.01, 1.02)	< 0.001
DBP (mmHg)	1.03 (1.02, 1.03)	< 0.001
BMI (kg/m^2^)	1.07 (1.06, 1.08)	< 0.001
FBG (mmol/L)	1.29 (1.24, 1.34)	< 0.001
CSe (μmol/L)	1.93 (1.57, 2.38)	< 0.001
Se intake (μg/d)	1.00 (1.00, 1.00)	0.593
Q1 (< 74.55)	Ref	
Q2 (74.55–107.20)	1.03 (0.87, 1.22)	0.716
Q3 (107.20–151.05)	1.31 (1.11, 1.54)	0.002
Q4 (≥ 151.05)	1.47 (1.25, 1.73)	< 0.001
Energy intake (kal/d)	1.00 (1.00, 1.00)	< 0.001
Q1 (< 1313.95)	Ref	
Q2 (1313.95–1807.00)	1.11 (0.95, 1.30)	0.181
Q3 (1807.00–2367.50)	1.29 (1.11, 1.50)	0.001
Q4 (≥ 2367.50)	1.75(1.50, 2.03)	< 0.001

FPRI, family poverty ratio of income; CHD, coronary heart disease; TC, total cholesterol; LDL, low-density lipoprotein; SBP, systolic blood pressure; DBP, diastolic blood pressure; BMI, body mass index; FBG, fasting blood glucose; CSe, circulating selenium; OR, odds ratio; CI, confidence interval.

### Multivariate logistic regression analysis of the relationship of CSe and arterial stiffness

Table 3 shows the relationship between CSe and AIP in different adjusted models. In Model 1, we observed that the AIP size increased as the CSe concentration rose (OR = 1.93, 95% confidence interval [CI]: 1.57, 2.38; *P* < 0.001) with no variables adjusted. In Model 2, the association between CSe and high AIP was positive (OR = 1.68, 95% CI: 1.37, 2.07; *P* < 0.001) with age, sex, and race adjusted. In Model 3, the relationship between CSe and high AIP was still positive (OR = 1.48, 95% CI: 1.20, 1.82; *P* = 0.001) with further adjustment. Additionally, after adjusting for all covariates, the positive association between CSe and high AIP was not changed in the highest CSe quartiles compared with the reference group (OR = 1.58, 95% CI: 1.30, 1.93, *P* < 0.001). Moreover, the trend for different CSe quartiles was also significant (*P* < 0.001). The relationship between CSe and high ePWV was also explored, as shown in Supplementary Digital Content, Table S3, available at: http://links.lww.com/JS9/F17. However, no correlation between CSe and high ePWV was noted in both the unadjusted and adjusted models.

**Table 3:** Multivariate logistic regression analysis of the relationship of CSe and high AIP

	Model 1		Model 2		Model 3	
Variable	OR (95% CI)	*P* value	OR (95% CI)	*P* value	OR (95% CI)	*P* value
CSe (μmol/L)	1.93 (1.57, 2.38)	< 0.001	1.68 (1.37, 2.07)	< 0.001	1.48 (1.20, 1.82)	0.001
CSe quartiles (μmol/L)						
Q1 (< 2.25)	Ref		Ref		Ref	
Q2 (2.25–2.44)	1.24 (1.02, 1.50)	0.031	1.17 (0.96, 1.43)	0.121	1.16 (0.93, 1.45)	0.198
Q3 (2.44–2.63)	1.45 (1.22, 1.72)	< 0.001	1.35 (1.13, 1.61)	0.002	1.26 (1.01, 1.57)	0.048
Q4 (≥ 2.63)	2.04 (1.73, 2.40)	< 0.001	1.81 (1.52, 2.15)	< 0.001	1.58 (1.30, 1.93)	< 0.001
*P* for trend		<0.001		< 0.001		< 0.001

CI, confidence interval; CSe, circulating selenium; FBG, fasting blood glucose; OR, odds ratio. Model 1 represents a rough analysis without any variable adjustment. Model 2 is a slightly corrected analysis, with adjustments made for three variables: age, sex, and race. Model 3 was further analyzed through correction, adjusting for age, sex, race, education, marital status, smoking, drinking, hypertension, diabetes, CHD, stroke, antihypertensive drugs, antidiabetic drugs, TC, LDL, SBP, DBP, BMI, FBG, Se intake, and energy intake.

### Dose-response relationship between CSe levels and arterial stiffness

**Figure 1.**
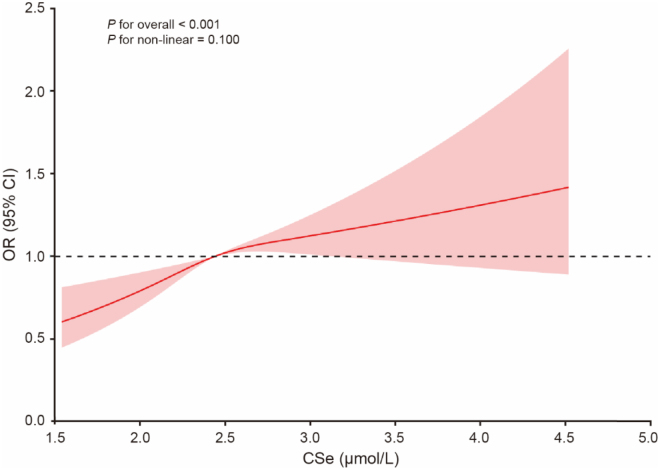
Dose-response relationship between CSe levels and high AIP based on restricted cubic splines. Red solid lines represent the estimates of OR for risks of high AIP, while shadows represent the corresponding 95% CI. CSe, circulating selenium; OR, odds rate; CI, confidence interval.

To detect the dose-response effect between the CSe levels and high AIP, we applied restricted cubic splines, as shown in Fig. 1. We discovered a positive linear relationship between the CSe index and a high AIP (*P* for nonlinearity = 0.1). Similarly, a dose-response relationship between the CSe index and high ePWV was observed with restricted cubic splines, as shown in Supplementary Digital Content, Fig. S2, available at: http://links.lww.com/JS9/F16. We discovered that the CSe levels were related to high ePWV in a U-shaped pattern (*P* for nonlinearity < 0.001). Furthermore, the inflection point was calculated using threshold effect analysis. While CSe levels < 2.99 μmol/L, CSe levels were negatively related to high ePWV (OR = 0.71; 95% CI: 0.59, 0.86; *P* < 0.001). In contrast, while CSe levels ≥ 2.99 μmol/L, CSe levels were positively related to high ePWV (OR = 2.66; 95% CI: 1.66, 4.25; *P* < 0.001).

### Subgroup analysis of the relationship between CSe levels and arterial stiffness

**Figure 2.**
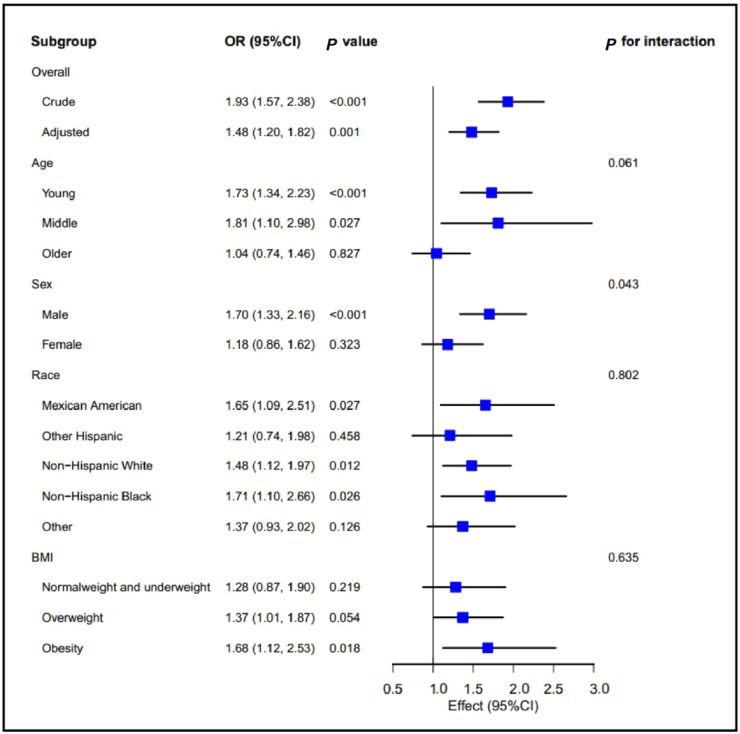
Subgroup analysis of the relationship between CSe levels and high AIP. BMI, body mass index; OR, odds rate; CI, confidence interval.

As shown in Fig. 2, the interaction effect of the correlation between CSe levels and high AIP was significant for sex (*P* = 0.043), whereas age, race, and BMI were not significant for interaction effects (all *P* > 0.05). Similarly, the interaction effect of the correlation between CSe levels and high ePWV was not significant (all *P* > 0.05), as shown in Supplementary Digital Content, Fig. S3, available at: http://links.lww.com/JS9/F16. Almost no positive relationship was observed between CSe levels and high ePWV.

### Mediation analysis

**Figure 3.**
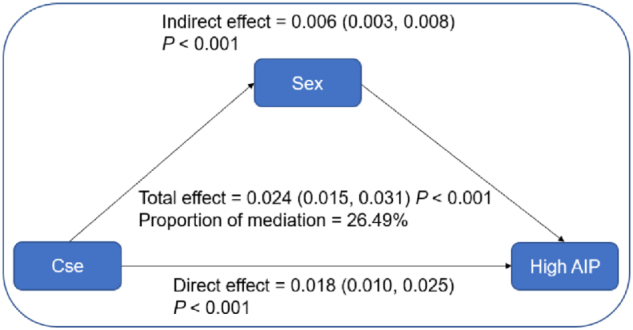
Mediation analysis of sex in the association of CSe with high AIP. CSe, circulating selenium; AIP, atherogenic index of plasma.

To better understand the relationship among sex, CSe, and high AIP, a mediation analysis was performed to examine the direct and indirect effects of CSe on high AIP. As shown in Fig. 3, CSe demonstrated a significant total effect on high AIP (total effect = 0.024; 95% CI: 0.015, 0.031). The indirect effect of sex on high AIP was prominent (indirect effect = 0.006; 95% CI: 0.003, 0.008; percent mediation = 26.49%) in adjusted Model 3.

### Sensitivity analyses

To verify the stability of the results, we analyzed AIP as a continuous variable in the univariate and multivariate regression analyses, and none of the results changed (Supplementary Digital Content, Tables S4 and S5, available at: http://links.lww.com/JS9/F17). After adjusting for all significant covariates, the AIP value increased with the increase of CSe (β = 0.06, 95% CI: 0.03, 0.08; *P* < 0.001). Additionally, compared with the lowest quartiles, CSe was positively related to AIP (β = 0.07, 95% CI: 0.04, 0.09, *P* < 0.001). The trend for the different CSe quartiles was also significant (*P* < 0.001). To further verify the stability of the results, we also analyzed the completed data before interpolation, and the results showed almost no change (Supplementary Digital Content, Tables S6 − S8, available at: http://links.lww.com/JS9/F17 Supplementary Digital Content, Figs S4−S6, available at: http://links.lww.com/JS9/F16). Therefore, our analysis results are robust.

## Discussion

Based on the above statistical analysis, we identified significant correlation among CSe, AIP, and ePWV as markers of arterial stiffness. The CSe was independently associated with an increased AIP in fully adjusted models. Furthermore, there was a U-shaped correlation with CSe and a high ePWV. In other words, CSe and high ePWV were negatively correlated on the left side of the inflection point and positively correlated on the right side of the inflection point. In addition, we found that sex interacted with the relationship between CSe and high AIP. The mediation analysis revealed that sex was important in this relationship.

Our results are inconsistent with those of previous studies. A study that included 106 hospitalized patients revealed that patients with more serious atherosclerosis, defined by arteriography, had lower plasma Se levels^[28]^. A cross-sectional study included 20–60-year-old adults residing in India and revealed no differences among different groups of serum Se levels based on cfPWV grades^[29]^. In addition, a prospective study that included 987 black adults in South Africa observed that cfPWV reduced as the concentration of serum Se elevated; however, the trend was not prominent^[30]^. A study of 988 participants aged 25–79 years in Luxembourg revealed that Se intake was negatively correlated with PWV in both sexes^[31]^.

ePWV is calculated using blood pressure and thus carries blood pressure information. A cross-sectional analysis of 2638 adults aged ≥ 40 years who were residing in the United States revealed that serum Se levels were positively correlated with SBP, DBP, pulse pressure, and hypertension^[32]^. A German study included 792 participants and revealed that serum Se concentration was positively related to SBP, DBP, pulse pressure, and hypertension and showed a “U” shape^[33]^. A longitudinal study of 2000 individuals aged ≥ 65 years from four rural areas in China revealed that as the concentration of nail Se increased, blood pressure levels and hypertension rates increased^[34]^. A cross-sectional survey including 9076 individuals aged 18–80 years and residing in Shandong, China, revealed a completely different outcome between the two sexes^[35]^. The U-shaped relationship between serum Se and ePWV implies that both excessive and insufficient Se are harmful to health. The U-shaped relationship may be attributed to the dual role of Se as both an essential micronutrient and a potential toxicant at extreme concentrations^[36]^. At moderate levels, Se exerts protective effects on vascular function through its incorporation into selenoproteins such as glutathione peroxidases (GPx), which reduce OS and inflammation—key contributors to arterial stiffness^[37,38]^. For instance, GPx-3 activity, a Se-dependent enzyme, was inversely correlated with vascular stiffness in clinical studies^[39]^. However, at very low Se levels, insufficient selenoprotein synthesis impairs antioxidant defenses, leading to elevated oxidative damage and accelerated vascular aging^[39–41]^. Furthermore, excessive Se intake (>300 µg/day) may paradoxically increase OS via pro-oxidant effects of inorganic Se species or disruption of selenoprotein homeostasis, as evidenced by an inverted U-shaped relationship with metabolic abnormalities^[42–44]^. Collectively, these mechanisms suggest that optimal Se status—neither deficient nor excessive—is critical for maintaining vascular elasticity, as reflected in the U-shaped ePWV relationship.

AIP is calculated using TG and HDL levels; therefore, AIP reflects blood lipid information. Abnormal blood lipid levels are risk factors for atherosclerosis. However, the results of studies on the relationship between Se and lipids have been inconsistent. An observational study of 2903 US adults revealed that CSe had a positive relationship with TC, LDL, and HDL levels^[45]^. A cross-sectional study of 1235 young Finns aged 3–18 years suggested that elevated serum Se levels were consistently correlated with elevated TC, HDL, and LDL levels^[46]^. A double-blind placebo-controlled randomized clinical trial included 60 Iranian patients diagnosed with atherosclerosis and revealed that 200 µg/day Se-enriched yeast for 8 consecutive weeks significantly reduced LDL levels compared with the placebo group, while the levels of TC, TG, and HDL did not decrease^[47]^. However, in a randomized trial including 3411 individuals in rural China, a mixture supplement of Se and vitamins that lasted for 7 years slightly increased the levels of TC and LDL, whereas HDL levels were not altered ^[48]^.

In subgroup analysis and mediating analysis, we found that sex played an important interactive and mediating role. In many ways, there are sex differences, such as sex hormones, life behaviors, and diseases^[49,50]^. The difference between Se and disease in different sexes may affect this effect. This sex disparity extends to disease risk, where Se shows an opposing relationship with the prevalence of diabetes—a positive correlation in females but a negative correlation in males^[40,51]^. A cross-sectional study in Europe revealed that a significant association between plasma Se and metabolic syndrome was found only among females^[52]^. The ePWV formula shows that this indicator carries hypertension information. Therefore, the sex difference of Se and hypertension can also affect this effect. The risk of hypertension shows sex-different Se interactions, with a particularly significant protective effect on males^[53]^. The study showed that the differences between the Se species and the biological markers of OS were linked to females, and the correlation between males was significantly greater compared to females^[54]^. The OS plays a significant role in the pathological mechanism of cardiovascular diseases^[21]^. Therefore, the relationship between Se and ePWV may be more significant in males than in females. This is consistent with the results of our subgroup analysis. Notably, the relationship between Se species and OS biomarkers was considerably stronger in males than in females^[54]^, suggesting sex-dependent redox regulation. Furthermore, the excess mortality risk related to Se deficiency was more than twice as strong in males compared to females^[55]^, indicating potential sex differences in Se utilization for cardioprotection.

The role of sex is particularly important in intervention studies. In the study of supplementing Se and coenzyme Q10 to intervene in the CVD mortality of the elderly group, it was found that the benefits for females seemed to be more obvious^[56]^. This difference may be related to the different Se requirements among different genders or the different pathogenesis of CVD. Interestingly, in diabetic patients, sex-specific alterations in NRF2 gene variations have modified the correlation between plasma Se and the risk of CVD^[57]^, emphasizing the complex interactions between sexes, genetics, and Se metabolism.

The synthesis and expression of Se in different sexes may affect this effect. The sex effects may stem from the differences in micronutrient metabolism and selenoprotein expression. The synthesis and expression of selenoenzymes and selenoproteins are related to sex differences^[58]^. Variations in the selenoprotein S1 gene contribute to cardiovascular risk only in females^[59]^. This metabolic difference may be influenced by the estrogen signaling, as suggested by the sex-specific gene expression patterns in selenoproteins^[60]^. Females have a higher Se content due to these differences^[61]^. This might make the effect of each unit change in Se on diseases more pronounced in males. In addition, the different proportions of smoking and drinking among different sexes also affect this mediating effect, as blood Se levels are higher in both drinkers and nonsmokers^[62]^.

These findings collectively demonstrate that sex acts as a critical mediator in the relationship through multiple biological and metabolic pathways. Future studies should explicitly account for sex differences when investigating the cardiovascular effects of Se. The development of sex-specific Se reference ranges and intervention strategies may optimize cardiovascular prevention.

This research has some advantages. Up to now, this research is the first to comprehensively demonstrate the relationship between CSe levels and arterial stiffness in the population representing the whole country. Furthermore, we explored the dose-response relationship and mediation of the association between CSe levels and arterial stiffness.

Nevertheless, several disadvantages also exist. First, because our analysis was based on a cross-sectional observational survey, the relationship between CSe level and arterial stiffness was correlated rather than causal. Second, although we adjusted for many important covariates, including Se intake, there may still be some potential confounding factors that have not been adjusted. Third, the arterial stiffness measurement used in our study was replaced by a standardized estimate rather than a gold standard measurement. Finally, the population in our study was from the US, which may have affected the extrapolation of the results.

## Conclusions

Our research demonstrated a notable correlation between CSe levels and arterial stiffness. It provides a certain reference value for Se supplement in the prevention, treatment, and health management of cardiovascular diseases, especially arteriosclerosis, in clinical practice. The cross-regional, multicenter, prospective, large cohort studies and randomized controlled trials are worthy of further research in the future.

## Data Availability

The data presented in this study is included in the article/Supplementary material, further inquiries can be directed to the corresponding authors.
